# Simultaneous saccharification and fermentation for d-lactic acid production using a metabolically engineered *Escherichia coli* adapted to high temperature

**DOI:** 10.1186/s13068-024-02579-1

**Published:** 2024-11-02

**Authors:** Gilberto Pérez-Morales, Luis Caspeta, Enrique Merino, Miguel A. Cevallos, Guillermo Gosset, Alfredo Martinez

**Affiliations:** 1 Department of Cellular Engineering and Biocatalyst. Instituto de Biotecnología, Universidad Nacional Autónoma de México, Av. Universidad 2001, Col. Chamilpa, 62210 Cuernavaca, Morelos México; 2 Department of Molecular Microbiology, Instituto de Biotecnología, Universidad Nacional Autónoma de México, Av. Universidad 2001, Col. Chamilpa, 62210 Cuernavaca, Morelos México; 3 Program of Evolutionary Genomics, Centro de Ciencias Genómicas, Universidad Nacional Autónoma de México, Av. Universidad 2000, Col. Chamilpa, 62210 Cuernavaca, Morelos México

**Keywords:** Simultaneous saccharification and fermentation (SSF), d-Lactic acid, Adaptive laboratory evolution (ALE), Thermotolerant *Escherichia coli*

## Abstract

**Background:**

*Escherichia coli* JU15 is a metabolically engineered strain capable to metabolize C5 and C6 sugars with a high yield of d-lactic acid production at its optimal growth temperature (37 °C). The simultaneous saccharification and fermentation process allow to use lignocellulosic biomass as a cost-effective and high-yield strategy. However, this process requires microorganisms capable of growth at a temperature close to 50 °C, at which the activity of cellulolytic enzymes works efficiently.

**Results:**

The thermotolerant strain GT48 was generated by adaptive laboratory evolution in batch and chemostat cultures under temperature increments until 48 °C. The strain GT48 was able to grow and ferment glucose to d-lactate at 47 °C. It was found that a pH of 6.3 conciliated with GT48 growth and cellulase activity of a commercial cocktail. Hence, this pH was used for the SSF of a diluted acid-pretreated corn stover (DAPCS) at a solid load of 15% (*w*/*w*), 15 FPU/g-_DAPCS_, and 47 °C. Under such conditions, the strain GT48 exhibited remarkable performance, producing d-lactate at a level of 1.41, 1.42, and 1.48-fold higher in titer, productivity, and yield, respectively, compared to parental strain at 45 °C.

**Conclusions:**

In general, our results show for the first time that a thermal-adapted strain of *E. coli* is capable of being used in the simultaneous saccharification and fermentation process without pre-saccharification stage at high temperatures.

## Background

Lactic acid (2-hydroxypropanoic acid) is a weak organic acid with a chiral carbon atom that can exist in the l( +) and d(-) stereoisomers. Given the diverse applications, including bioplastics for packing, food industries, and medical-pharmaceutical applications, lactic acid (LAC) is considered one of the main chemicals from biomass with a near-term potential [1, 2]. Therefore, its market has been projected to grow at an annual rate of over 5% continuously [1]. Although, the market has been dominated by l-lactic acid (l-LAC), there is a growing interest in producing d-lactic acid (d-LAC) for polylactic acid manufacture, a biodegradable polymer that requires optically pure d-LAC to obtain proper thermomechanical properties [3, 4].

Currently, LAC is mainly produced by fermentation of glucose from starch and sucrose from sugarcane [5]. However, the extensive repertoire of applications pursuing LAC production in recent and coming years has been increasing the interest in obtaining this organic acid from a more abundant feedstock like lignocellulosic biomass [4, 6, 7]. Lignocellulosic biomass represents an attractive alternative carbon source for LAC fermentation since it does not compete with the food supply chain and contains, on average, around 60% of fermentable sugars [8]. However, releasing of these sugars has become one of the main bottlenecks for the economical production of chemicals and biofuels, since thermochemical pretreatment and saccharification are needed [9]. Saccharification represents the higher cost of these two unit operations, as nearly 50% of production costs are involved in the cellulosic enzymes needed to break cellulose down into glucose [10, 11]. Saccharification is often carried out before fermentation, at 50 °C and pH 5, for optimal activity of cellulases [12], these conditions are barely tolerated by industrial microorganisms and lactic acid bacteria. However, there is an increasing interest in performing simultaneous saccharification and fermentation (SSF) because this allows to proceed in one unit operation. Furthermore, SSF reduces production costs because it diminishes cooling efforts, enzyme inhibition by released glucose, and contamination [13].

Different microorganisms have been studied to produce LAC from SSF of lignocellulosic biomass. Wild-type microorganisms such as lactic acid bacteria (*Bacillus coagulans*, *Pediococcus acidilactici* and *Lactobacillus sp.*) have demonstrated their potential to obtain high titers of LAC production. Furthermore, the genetic engineering of these microorganisms has allowed to improve the production of LAC to increase optical purity, consumption of sugars and its homofermentative capacity [14]. The production of LAC under SSF conditions has been previously reported using strains of *B. coagulans* because it has the advantage of growing at a temperature of 50 °C [15–18]. Additionally, a strain of *L. bulgaricus* has been evolved to increase its thermotolerance to produce LAC by SSF [19]. However, the use of these microorganisms that synthesize LAC naturally are challenged by the high cost of nutritional requirements, the assimilation of few sugars, the low tolerance of inhibitory compounds, the low optical purity of LAC and the sensitivity to acidic pH [14, 20]. Although there are other promising genetically modified microorganisms like *Klebsiella oxytoca* [21, 22] and *Kluyveromyces marxianus* [23] that have been developed to produce optically pure d-LAC at high titers, the main drawback is that they do not thrive at the temperature range of cellulases cocktails used for SSF (45–55 °C) [24].

Metabolic engineering has been used to generate LAC producing strains of *E. coli*. For instance, the very low yield of lactate production by the wild-type *E. coli* strain MG1655 (0.083 g/g of glucose) was overcome by eliminating competitive reactions for lactate synthesis to generate the CL3 strain which produced d-LAC with a yield of 0.95 g/g of glucose [25]. By deleting *xylFGH* genes and using adaptive laboratory evolution, the strain CL3 was evolved to strain JU15, which kept high lactate accumulation while consuming xylose at a faster rate [26]. This strain produced d-lactate (d-LAC) efficiently with titers between 40 and 60 g/L and yields close to 0.90 g/g of glucose and/or xylose from thermochemical-enzymatic hydrolysates of sugarcane bagasse, corn stover, avocado seeds, and food waste with minimal nutrient addition [27–29]. Since *E. coli* strains barely grow efficiently at temperatures higher than 44 °C [30] and at pH values lower than 5 [31], the benefits of using strain JU15 in SSF are limited.

Adaptive laboratory evolution (ALE) is a metabolic engineering strategy that allows obtaining strains with an improved and desired phenotype, based on the natural adaptation processes challenged by a specific selective pressure [22, 26, 32]. Many of the ALE experiments reported in the literature have dealt with the generation of *E. coli* strains with improved metabolic capability to consume better carbon sources found in agro-industrial wastes, including xylose, cellobiose, lactose, and glycerol [4, 26, 33]. ALE experiments have also been applied for generating strains capable of growing at temperatures close to 48 °C in rich culture media [34, 35], and to increase the tolerance to alcohols like ethanol, isobutanol and n-butanol [33].

In this study, we used ALE experiments in batch and chemostat cultures to generate a thermotolerant strain from the homolactic ancestor JU15, to develop SSF processes to transform the cellulosic fraction of diluted acid-pretreated corn-stover (DAPCS) biomass into d-LAC efficiently. The main result shows that SSF can be performed with the evolved strain at 47 °C, pH 6.3, and using a solid load of 15% (*w*/*w*), reaching a titer of 59 g/L and a yield of 71% of d-LAC from the glucan fraction presented in the solid fraction of DAPCS.

## Materials and methods

### Organisms and media

The *E. coli* strain JU15 (MG1655: Δ*pflB*, Δ*frdA*, Δ*adhA* Δ*xylFGH*, Δreg 27.3 kpb, GatC^S184L^) [26, 27] was used for the Adaptive Laboratory Evolution experiments, resulting in the thermal-adapted endpoint strain *E. coli* GT48. Both, the parental strain and the strain GT48 were evaluated in 300-mL mini-fermenters using minimal medium supplemented with glucose. The minimal medium AM1 was composed of 2.63 g/L (NH_4_)_2_HPO_4_, 0.87 g/L NH_4_H_2_PO_4_, 1.5 mM MgSO_4_·7H_2_O, 1.5 mL/L trace metal solution, 2.0 mM KCl and 1.0 mM betaine [36]. Trace element solution contained per liter: 1.6 g FeCl_3_, 0.2 g CoCl_2_·6H_2_O, 0.1 g CuCl_2_, 0.2 g ZnCl_2_·4H_2_O, 0.2 g Na_2_MoO_4_, 0.05 g H_3_BO_3_ and 0.33 g MnCl_2_·4H_2_O. The culture medium was supplemented with 0.1 g/L of sodium citrate, 40 g/L of glucose, 0.1 or 1 g/L tryptone, and 0.05 or 0.5 g/L yeast extract (0.15 or 1.5 g/L of protein hydrolysates), as described in the results.

### Adaptive laboratory evolution experiments

The adaptive laboratory evolution (ALE), from 37 °C to temperatures lower than 45 °C, was carried out through two distinct cultivation strategies. The first strategy was performed in batch cultures by serial transfers in 300 mL home-made mini-fermenters with 200 mL working volume at 150 rpm of agitation, without aeration, controlling the pH at 7 by automated addition of 2 N KOH, and using the medium described above without tryptone and yeast extract. From a conserved cryovial of parental strain JU15, 5 mL of LB medium was inoculated at 37 °C. Once the culture reached cell saturation, a mini-fermenter was inoculated under ALE conditions at 37 °C. Throughout the ALE, serial passages were performed once the cultures reached the exponential phase to initiate a new batch culture with an initial optical density at 600 nm (OD_600_) ranging from 0.05 to 0.1 (Spectrophotometer Genesys 10S UV–vis, Thermo Scientific, Waltham, MA). During batch ALE, the temperature was gradually increased by 2 °C, 1 °C, or 0.5 °C once the culture showed a growth rate close to the maximal at 37 °C. When reaching 45 °C during the ALE the medium was supplemented with 0.1 g/L tryptone and 0.05 g/L yeast extract to support the adaptation process.

The second ALE strategy was carried out in a chemostat regimen [37] using a 1-L bioreactor (Applikon ADI 1010—Ez-control, Delft, NL). The working volume was set to 750 mL, the agitation was controlled at 400 rpm, and the pH was automatically controlled to neutral levels by adding 8 N KOH. Cell culture startup was initiated at 0.1 OD_600_ (0.037 g-_DCW_/L) in batch mode without aeration at 45 °C. After reaching the stationary phase, the continuous culture operation was initiated by flowing 1.125 mL/min of a concentrated medium, corresponding to a dilution rate (D) of 0.09 h^−1^. During the ALE chemostat process, the temperature was gradually increased by 0.2 °C after three residence times at the steady state. This increase in the fermentation temperature prevents detrimental exposure to the cell because at temperatures greater than 43 °C increments higher than 0.2 °C drastically reduced the cell viability. For temperatures ranging from 46.5 °C to 48 °C, the tryptone and yeast extract content was increased to 1 g/L and 0.5 g/L, respectively. The cell concentration was maintained within the initial interval values. Prior to each temperature increment, 0.8 mL of sample were taken, mixed with 80% glycerol, and stored at − 70 °C. The endpoint strain was obtained by selecting and evaluating colonies isolated from the cryovial-preserved sample.

### Batch cultivations for strain evaluation

Batch cultures were performed using AM1 minimal medium supplemented with 0.1 g/L of sodium citrate, 40 g/L of glucose, 1 g/L tryptone and 0.5 g/L yeast extract. The cells were cultivated in 300-mL mini-fermenters [38], each containing 200 mL of culture medium, operating without aeration at 150 rpm, and an initial inoculum of 0.1 OD_600_. The temperature was controlled with a thermocirculator, while pH was maintained constant (either at 7.0 or 6.3, as indicated in the results) with the automatic addition of a 2 N KOH solution. All experiments were carried out in triplicate.

### Cultivations with non-growing but metabolically active cells

Batch cultivations with non-growing but metabolically active cells (NGMAC) at 48 °C were additionally conducted by a two-phase cultivation mode using AM1 minimal medium supplemented with 0.1 g/L of sodium citrate, 40 g/L of glucose, 1 g/L tryptone and 0.5 g/L yeast extract. In the first cultivation phase, the cells were cultivated in batch cultures with an initial inoculum of 0.1 OD_600_, 47 °C, and pH 7 in the same way described in the preceding paragraph. The second cultivation phase was conducted at 48 °C and pH 7 with an initial inoculum size of 1 g-_DCW_/L of cells harvested at the mid-exponential phase from the batch cultivation performed at 47 °C. This experiment was carried out in triplicate.

### Pretreatment of corn stover biomass

The corn stover was pretreated by a thermo-acid pretreatment process implemented by [39]. Briefly, dry solids of corn stover were milled with a hand mill and sieved (with mesh numbers of 20 and 80) to obtain a mixture of particles with sizes between 1–2 cm. This material was soaked in 1% H_2_SO_4_ solution to obtain a slurry with 15% (w/w) solids, which was heated, in a Parr reactor (Parr, Moline, IL. USA), until reaching 130 °C and maintained at this temperature for 30 min. The suspension was cooled, and the solids were separated and washed with distilled water. The pH of the dissolved solids in water was adjusted to 6.3 with a 2 N KOH solution, rewashed with distilled water, the water was removed, and the solids were dried at 50 °C.

### Enzymatic saccharification

For comparison, with the simultaneous saccharification and fermentation, a set of experiments comprising only enzymatic saccharification were performed with 15% (w/w) solid loading of a diluted acid-pretreated corn stover (DAPCS) biomass using enzyme dosages of 15 FPU/g_-DAPCS_ from Cellic^®^ CTec2 (Novozymes, Bagsværd, Denmark). The process was operated at temperatures of 47 °C and 50 °C and a pH of 6.3 in 300-mL mini-fermenters with peg-mixers [40]. The AM1 mineral medium supplemented with 0.1 g/L sodium citrate, 1 g/L tryptone, 0.5 g/L yeast extract, and ammonium phosphate ((NH_4_)_2_HPO_4_ and NH_4_H_2_PO_4_,), adjusted at a pH 6.3 was used as a buffer, and to have the same liquid composition used in the simultaneous saccharification and fermentation studies. The activity of the enzymatic cocktail Cellic^®^ CTec2 (Novozymes) was assessed for filter paper unit (100 ± 1.01 FPU/mL) using filter paper as substrate as described by Ghose [41], and the protein concentration (176.28 ± 0.99 mg of protein/mL) was determined using the Quick Start^™^ Bradford Protein Assay (Bio-Rad, Hercules, CA). Finally, filter paper activity per milligram of the total protein in the commercial cellulase cocktail resulted in 0.57 ± 0.003 FPU/mg of protein. All experiments were carried out in triplicate.

### Simultaneous saccharification and fermentation of a diluted acid-pretreated corn stover biomass

Simultaneous saccharification and fermentation (SSF) were carried out in a 300-mL mini-fermenters system agitated with peg-mixer type impellers [40]. The pH was controlled at 6.3 by the automatic addition of 2 N KOH. SSF with the parental JU15 strain was evaluated at 45 °C and the evolved strain GT48 was assessed at 47 °C. The SSF was performed at different solid loadings of DAPCS (g_-DAPCS_) and enzyme dosages (FPU/g_-DAPCS_: filter paper unit per gram of a diluted acid-pretreated corn stover biomass) for 48 h. Particularly, three enzyme dosages were evaluated at 5, 10 and 15 FPU/g_-DAPCS_, corresponding to 8.77, 17.54, and 26.32 mg of protein_cocktail_/g_-DAPCS_, respectively. The solids were dissolved in AM1 mineral medium supplemented with 0.1 g/L sodium citrate, 1 g/L tryptone, and 0.5 g/L yeast extract in a 200 mL working volume. The amount of ammonium phosphate ((NH_4_)_2_HPO_4_ and NH_4_H_2_PO_4_) in AM1 was adjusted to obtain a phosphate buffer with a pH of 6.3. The cells from the inoculum were harvested at the mid-exponential phase from batch cultivations performed at 47 °C, to reach an inoculum size of 1 OD_600_. The cells and the enzyme cocktail Cellic^®^ CTec2 (Novozymes) were added simultaneously, at the very beginning of the SSF. These experiments were performed in duplicate.

### Analytical methods

The cell concentration was determined indirectly by the optical density at 600 nm with a spectrophotometer Genesys 10S UV–vis (Thermo Scientific, Waltham, MA) and converted to mass in dry cell weight (DCW) per liter using the following relationship: 1 optical density (OD_600_) = 0.37 g_-DCW_/L. After measuring the optical density, the samples were immediately centrifuged with a centrifuge Sorvall ST 16/16R (Thermo Scientific, Waltham, MA), and the supernatants were stored at − 20 ° for further analysis. High-performance liquid chromatography (HPLC) (Waters, MA, USA) was used to determine the concentration of glucose and d-LAC in the supernatant. The separation of metabolites was performed using the Aminex HPX-87H column (Bio-Rad, Hercules, CA) operated at a temperature of 60 °C. The mobile phase consisted of a 5 mM H_2_SO_4_ solution, which was flowed at 0.5 mL/min. The compounds were detected with a diode array detector (Waters 996, MA, USA) and a refractive index detector (Waters 410, MA, USA). The glucose and d-LAC concentrations were calculated using a calibration curve obtained with pure standards.

### Statistical analysis

Depending on the case, the difference in kinetic and stoichiometric parameters was compared using the Student’s t-test or ANOVA one-way. A *p*-value of < 0.05 was considered statistically significant.

## Results and discussion

### A combination of different ALE strategies generated the thermotolerant *E. coli* strain GT48

High-temperature adaptive laboratory evolution (ALE) experiments were performed using the parental strain JU15, two distinct cultivation strategies, and the medium described in the methods section with 40 g/L glucose at pH 7 and without aeration. The initial strategy involved serial transfers in batch cultures starting at 37 °C. Upon reaching a temperature of 45 °C, during the ALE batch experiments, the culture medium was supplemented with 0.1 g/L tryptone and 0.05 g/L yeast extract to facilitate the adaptation process of strain JU15 to a temperature up to 45 °C. Subsequently, the second ALE strategy entailed a chemostat culture initiated at 45 °C from the single colony obtained from the previous strategy. Throughout the adaptation process from 45 °C to 46.5 °C the composition of the culture medium remained unchanged. Finally, as temperatures exceeded 46.5 °C, the concentration of tryptone and yeast extract was increased to 1 g/L and 0.5 g/L, respectively, culminating in an operating temperature of 48 °C.

After 31 serial passages of batch cultures and 151 days of continuous culture of experimental evolution, the evolved population was sampled and cultivated in Petri dishes at 45 °C. From this population, six colonies were carefully selected, and subsequently cultured at 48 °C in mini-fermenters containing the glucose-minimal medium with 1 g/L tryptone and 0.5 g/L yeast extract. Despite the strains being acclimated at 45 °C before transferring them to 48 °C, none of the selected colonies exhibited growth during batch cultivations at elevated temperatures. Consequently, evolved strains were selected at 47 °C, where the parental strain JU15 could not thrive. In this condition, we selected the GT48 strain. The impossibility of keeping growth by a selected thermotolerant strain at the evolved temperature was also observed by Blaby et al. [34]. They evolved a population of wild-type *E. coli* under gradually increasing temperatures up to 49.7 °C. However, the isolated strains exhibited the ability to grow in batch cultivations at temperatures up to 48.5 °C in a nutrient-rich LB medium.

ALE experiments have been conducted to select thermotolerant *E. coli* strains in cultivations using minimum defined media, with temperatures incrementally increased up to 45 °C [42–45]. To enable temperatures exceeding 48.5 °C, the authors used nutrient-rich LB media [34, 35]. The rationale behind media supplementation at temperatures above 42 °C is that *E. coli* cannot synthesize methionine and certain cofactors due to the thermo-susceptibility of specific enzymes that limit some steps in the metabolic pathways [46, 47]. In this study, we supplemented some components of LB medium at reduced concentrations (1 g/L tryptone and 0.5 g/L yeast extract) in the AM1 mineral medium, as an alternative to nutrient-rich Luria broth, which typically contains 10 g/L tryptone and 5 g/L yeast extract.

### The thermal-adapted strain GT48 maintains the lacto-homofermentative capacity at 47 °C

To evaluate the physiological responses of the thermotolerant GT48, both GT48 strain and its progenitor strain JU15 were cultivated in glucose-minimal medium supplemented with 1 g/L tryptone and 0.5 g/L yeast extract at 37 °C and 47 °C (which represents a 10 °C increase above the optimum growth temperature for parental *E. coli* strains). At 37 °C, GT48 exhibited a growth rate that was slightly slower than JU15 (Table 1), with a slightly lower biomass yield from glucose as well, but similar specific glucose consumption and specific d-LAC production rates. At 47 °C, JU15 was not able to grow, while GT48 displayed only half of the specific growth rate observed at 37 °C. Moreover, the specific glucose consumption rate decreased by 10% for GT48 at 47 °C compared to 37 °C. Notably, at 47 °C GT48 exhibited similar volumetric productivity and d-LAC yield compared to 37 °C (Table 1).

**Table 1:** Fermentation parameters of strains JU15 and GT48 at 37 and 47 °C (pH 7)

Parameter	37 °C		47 °C	
	JU15	GT48	JU15	GT48
µ_exp_ (h^−1^)	0.65 (0.11)	0.63 (0.007)	–	0.32 (0.013) ^****^
X_24_ (g _DCW_/L)	1.11 (0.010)	0.86 (0.038) ^***^	–	0.81 (0.009)
Yp/s (g/g)	91.80% (0.63)	92.66% (1.57)	–	94.89% (6.97)
Yx/s (g/g)	7.72% (0.50)	7.38% (0.11)	–	4.20% (0.280) ^****^
qs (g/g∙h)	8.37 (0.400)	8.47 (0.028)	–	7.62 (0.814)
qp (g/ g∙h)	7.69 (0.315)	7.85 (0.159)	–	7.20 (0.242)
Qp_24_ (g/L∙h)	1.47 (0.044)	1.46 (0.011)	–	1.56 (0.001) ^****^

AM1 minimal medium was supplemented with 1 g/L tryptone and 0.5 g/L yeast extract. µ_exp_: specific growth rate. X_24_: biomass reached at 24 h. Yp/s: specific yield of d-LAC on glucose. Yx/s: specific biomass yield on consumed glucose. qs: specific glucose consumption rate. qp: specific d-LAC production rate. Qp_24_: volumetric productivity of d-LAC at 24 h. Values in parenthesis indicate standard deviation from triplicates. Asterisks represent the statistically significant differences from a Student’s *t*-test performed between: (a) GT48 and JU15 at 37 °C; and (b) GT48 at 47 °C and GT48 at 37 °C

It has been observed that glycolytic fluxes increased while the metabolic rate in TCA decreased with the concomitant synthesis and accumulation of d-LAC to regenerate NAD^+^ in an *E. coli* strain used for recombinant protein production [48]. Additionally, the expression of the d-lactate dehydrogenase gene increased 13-fold in *E. coli* MG1655 following exposure to elevated temperature [49]. It has been suggested that increasing the glycolytic activity serves as a mechanism to provide immediate ATP to deal with the increased demand for maintenance energy upon temperature increase [48, 50]. Together, these data suggest that the increase in d-LAC accumulation in GT48 cultivations at 47 °C compared with 37 °C could be correlated with a natural *E. coli* metabolic response to high temperature, as well as changes that occur in genome structure accounted by this strain to improve thermotolerance during evolution.

Most of the enzymes of the glycolytic and fermentation pathways, including LDH, are stable at temperatures as high as 50–60 °C according to BRENDA database [51]. This evidence and the fact that non-growing bacteria better controlled the ATP demand [52] and that GT48 remained viable at 48 °C, let us hypothesize whether GT48 kept proper metabolic activity at 48 °C. Therefore, we explored the potential use of this thermotolerant *E. coli* in processes with non-growing but metabolically active cells (NGMAC). Another hypothesis was the fact that lactate yield could increase since division does not occur in NGMAC and endogenous respiration is either absent or significantly reduced in this condition [53].

**Fig. 1:**
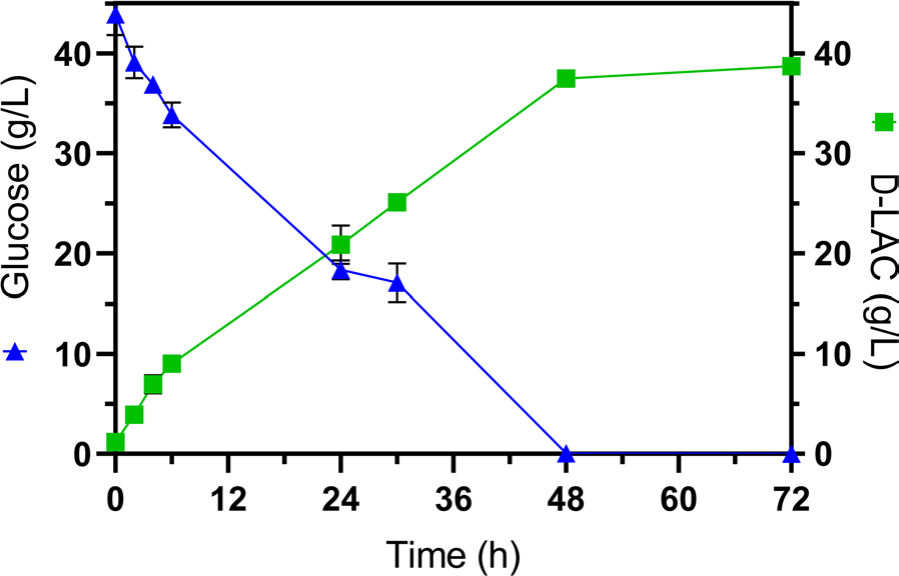
Bioconversion of 40 g/L glucose to d-LAC by NGMAC of thermotolerant strain GT48 at 48 °C and pH 7. Error bars in the figures represent the standard deviation from triplicates

As depicted in Fig. 1 and Table 2, NGMAC of GT48 efficiently utilized all available glucose present in the medium at 48 °C in 48 h, and the d-LAC was simultaneously accumulated until a concentration approaching 40 g/L. The highest d-LAC production and glucose consumption rates were observed in the first 24 h (0.82 and 1.06 g/L/h). However, these rates remained barely constant until 48 h (Fig. 1). Consequently, after 48 h of fermentation elapsed time, NGMAC of GT48 exhibited d-LAC accumulation rates that were approximately 40% slower than those observed in actively growing cells (Table 1). As a result, subsequent simultaneous saccharification and fermentation (SSF) studies with GT48 were performed at 47 °C. Accordingly, the activity of cellulosic enzymes was also tested at this temperature to ensure suitable saccharification under the chosen fermentation conditions. The enzymatic activity at 47 °C and pH 6.3 (54.67 ± 1.28 FPU/mL) was 42.3% lower than the optimal conditions of the enzyme cocktail (50 °C and pH 4.8) and only 5.3% lower compared to 47 °C and pH 6.

**Table 2:** Kinetic parameters of fermentation by NGMAC with strain GT48 at 48 °C (pH 7)

Parameter	Cultivation time (h)	GT48
Qs (g/L∙h)	24	1.06 (0.047)
	48	0.91 (0.042)
	72	0.61 (0.029)
Qp (g/L∙h)	24	0.82 (0.085)
	48	0.76 (0.002)
	72	0.52 (0.001)
Yp/s (g/g)	24	76.87% (4.64)
	48	82.83% (3.57)
	72	85.59% (3.83)

Qs: volumetric rate of glucose consumption. Qp: volumetric productivity of d-LAC. Yp/s: yield of d-LAC on glucose. Values in parenthesis indicate standard deviation from triplicates

### Cellulases initial saccharification rate at 47 °C maintains up to ~ 90% of the initial saccharification rate at 50 °C

**Fig. 2:**
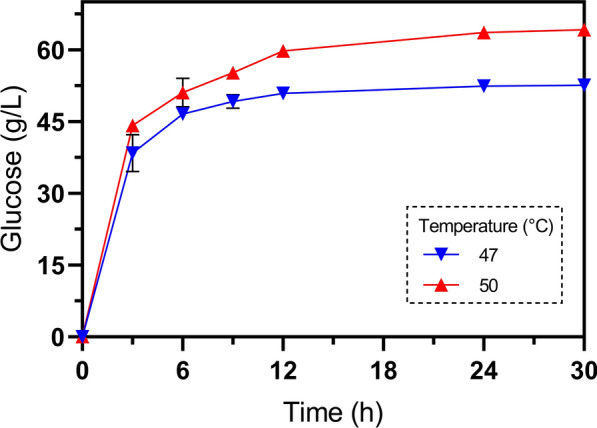
Enzymatic saccharification of 15% (*w*/*w*) diluted acid-pretreated corn stover with 15 FPU/g_-DAPCS_ and pH 6.3 at 47 °C and 50 °C. Error bars in the figures represent the standard deviation from triplicates

The optimum average temperature for saccharification is typically cited as 50 °C. However, given the superior growth and d-LAC production exhibited by strain GT48 at 47 °C, the next logical step in developing an SSF process was to assess saccharification using the Novozymes cocktail Cellic^®^ CTec2 at both temperatures. For these experiments, a solid loading of a diluted acid-pretreated corn stover of 15% (*w*/*w*) and an enzymatic load of 15 FPU/g-_DAPCS_ were evaluated, as these conditions have been shown to yield high concentrations of glucose [39]. Glucose accumulation under both 47 °C and 50 °C at a pH value of 6.3 (see below) is illustrated in Fig. 2. A slight difference in glucose accumulation was observed during the initial 6 h of saccharification at both temperatures. After 12 and 24 h, when the saccharification rate highly decreased at both temperatures, the process at 50 °C accumulated 21% more glucose than at 47 °C. For instance, after 24 h, glucose accumulation reached 64 g/L at 50 °C while only 53 g/L was observed at 47 °C. Considering that pretreated corn stover with 1% acid contains 51.5% of glucan on average [39], these results from saccharification at 47 °C and 50 °C represented efficiencies of 67% and 84% of glucose released from glucan after 48 h, respectively. Although enzymes typically experienced lower product inhibition in SSF, we anticipated that the observed 17% decrease in glucose accumulation at 47 °C could potentially be overcome during SSF.

In summary, it was determined that a temperature of 47 °C conciliated for both enzyme activity and GT48 growth. However, to perform SSF experiments effectively, it was also essential to assess d-LAC production at a pH value that aligns with the optimal activity of cellulolytic enzymes (pH 5) and GT48 growth (pH 6–7). This evaluation was assessed at 47 °C.

### The thermal-adapted GT48 strain exhibits a specific growth and glucose consumption rates at pH 6.3 comparable to those at pH 7

The growth of GT48 was evaluated at pH values of 6, 6.3, and 7, with results summarized in Table 3. Notably at pH 6.3 and 7, the specific growth and glucose consumption rates were similar. However, d-LAC accumulation rates and yields experienced a 15% decrease at pH 6.3 compared to pH 7. At pH 6, there was a significant reduction in growth and d-LAC accumulation rates by 50%, alongside a 24% decrease in d-LAC yield compared to pH 7. Since there was a small difference in cellulolytic activity between pH values of 6 and 6.3 at 47 °C (57.76 vs. 54.67 FPU/mL, respectively), and the growth performance of GT48 was reduced by 50% under this range of pH, we decided to use a pH of 6.3 in the following experiments of SSF.

**Table 3:** Fermentation parameters of strain GT48 in batch cultures at different pH values (47 °C)

Parameter	pH	
	6	6.3
µ_exp_ (h^−1^)	0.16 (0.006)^****^	0.33 (0.006)
X_24_ (g _DCW_/L)	0.47 (0.012)^****^	0.78 (0.016)
Yp/s (g/g)	76.60% (0.845)^*^	80.00% (1.360)
Yx/s (g/g)	2.24% (0.065)^***^	4.53% (0.504)
qs (g/g∙h)	7.12 (0.468)	7.40 (0.954)
qp (g/g∙h)	5.46 (0.419)^**^	5.85 (0.243)
Qp_24_ (g/L∙h)	0.80 (0.024)^****^	1.35 (0.063)

µ_exp_: specific growth rate. X_24_: biomass reached at 24 h. Yp/s: specific yield of d-LAC on glucose. Yx/s: specific biomass yield on glucose. qs: specific glucose consumption rate. qp: specific d-LAC production rate. Qp_24_: volumetric productivity of d-LAC at 24 h. Values in parenthesis indicate standard deviation from triplicates. Asterisks represent the significant statistical differences from a Student’s *t*-test performed between each pH versus pH 7. No significant differences were found between pH 6.3 and pH 7

### The thermal-adapted GT48 strain enables SSF at high temperature

**Fig. 3:**
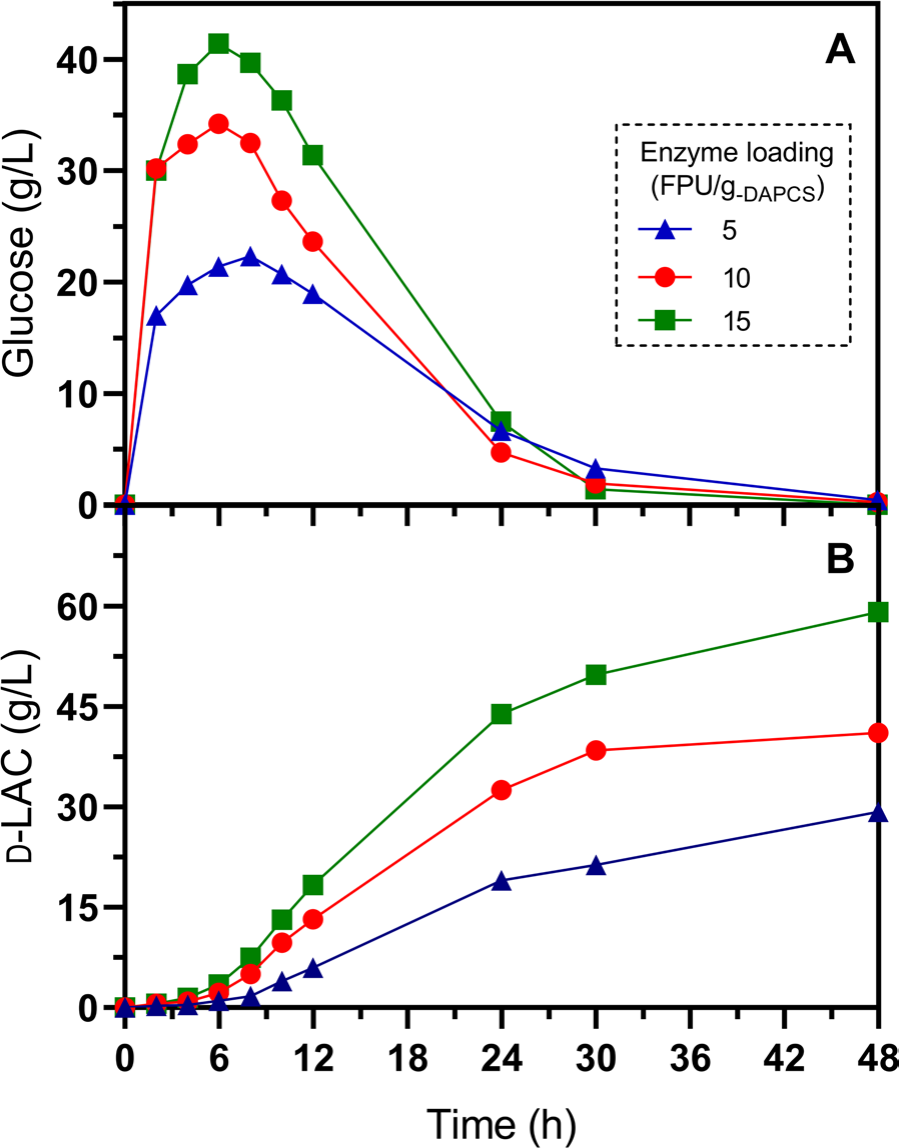
Simultaneous saccharification and fermentation of 15% (*w*/*w*) diluted acid-pretreated corn stover at different enzyme loadings, pH 6.3 and 47 °C. **A** Glucose release and consumption with an enzyme loading of 5, 10, and 15 FPU/g_-DAPCS_. **B** d-LAC production with an enzyme loading of 5, 10, and 15 FPU/g_-DAPCS_. Error bars in figures are shorter than the size of the symbol. These cultivations were performed in duplicate

The potential use of the homolactic, thermotolerant *E. coli* GT48 to produce d-LAC was evaluated in SSF experiments at 47 °C, pH 6.3, and 15% (*w*/*w*) solids loading of diluted acid-pretreated corn stover. Since one of the potential advantages of SSF is to diminish cellulases inactivation, caused by glucose inhibition, which results in a reduction of the amount of enzyme loading [24], we decided to test three enzymatic loadings: low (5 FPU/g_-DAPCS_), middle (10 FPU/g_-DAPCS_), and high (15 FPU/g_-DAPCS_). It is worth noting that the solids loadings, GT48 inoculum, and enzyme dosage were simultaneously added to the SSF process, eliminating the need for a pre-saccharification step before adding the inoculum to initiate glucose fermentation (Fig. 3).

**Fig. 4:**
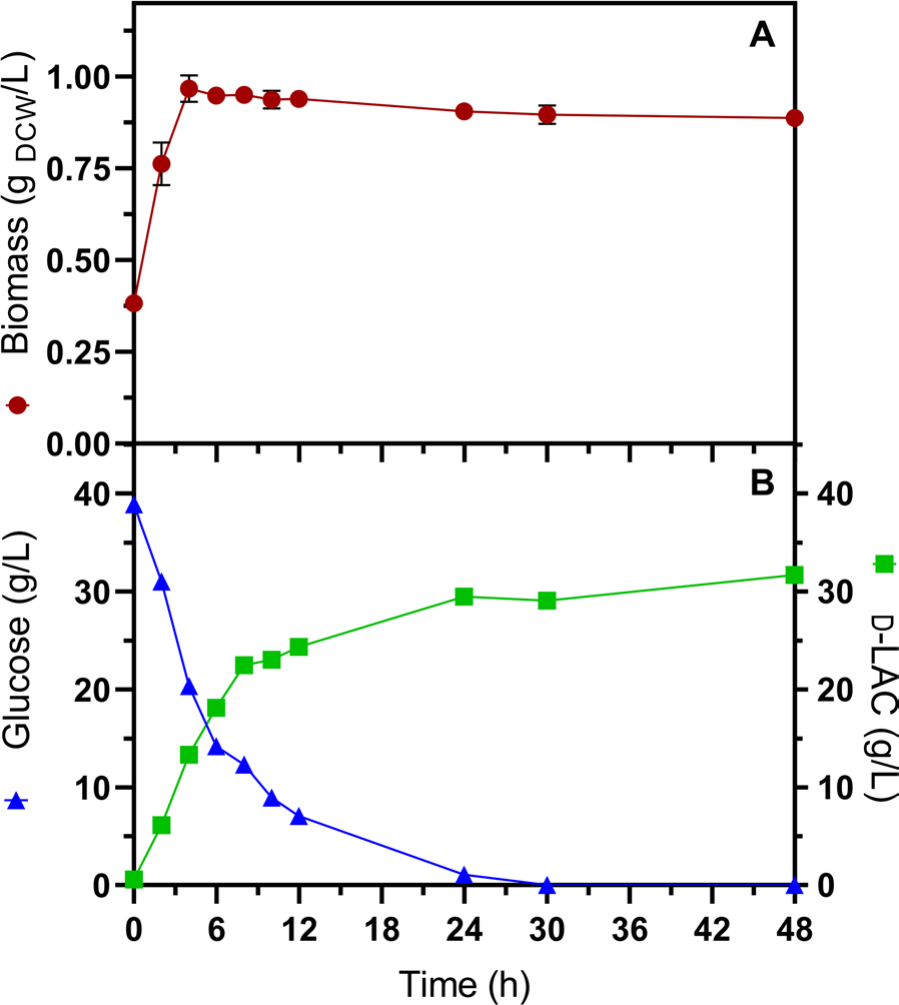
Control fermentation of 40 g/L glucose at 47 °C, pH 6.3, and 0.37 g-_DCW_/L of the initial inoculum. **A** Biomass, **B** glucose consumption, and d-LAC production. Error bars in the figures represent the standard deviation from triplicates

As can be seen in Fig. 3A, glucose was immediately accumulated, reaching concentrations of 42, 34, and 23 g/L after the first 6 h of SSF with 15, 10, and 5 FPU/g_-DAPCS_, respectively. Conversely, there was a negligible production of d-LAC during this same period. The amount of glucose released with 15 FPU/g_-DAPCS_ mirrors the accumulation observed during saccharification alone (Fig. 2). This implies a lag phase for activation of cellular metabolism during SSF, contrasting with the control experiment and d-LAC production in minimal-defined media supplemented with 1 g/L tryptone and 0.5 g/L yeast extract under identical culture conditions. In this setting, 18 g/L of d-LAC accumulated after 6 h (Fig. 4). Namely, in SSF processes, the lag period of cellular metabolic activity must be considered when adding the inoculum since this negatively impacts productivity. The extension of the lag phase resulting from the low glucose consumption and LAC production rate during SSF could be due to the decrease in cell viability by the solid’s load and inhibitory effects by the release of phenolic compounds [54], as well as mass transfer limitations for improper mixing that reduce process efficiency [24].

**Fig. 5:**
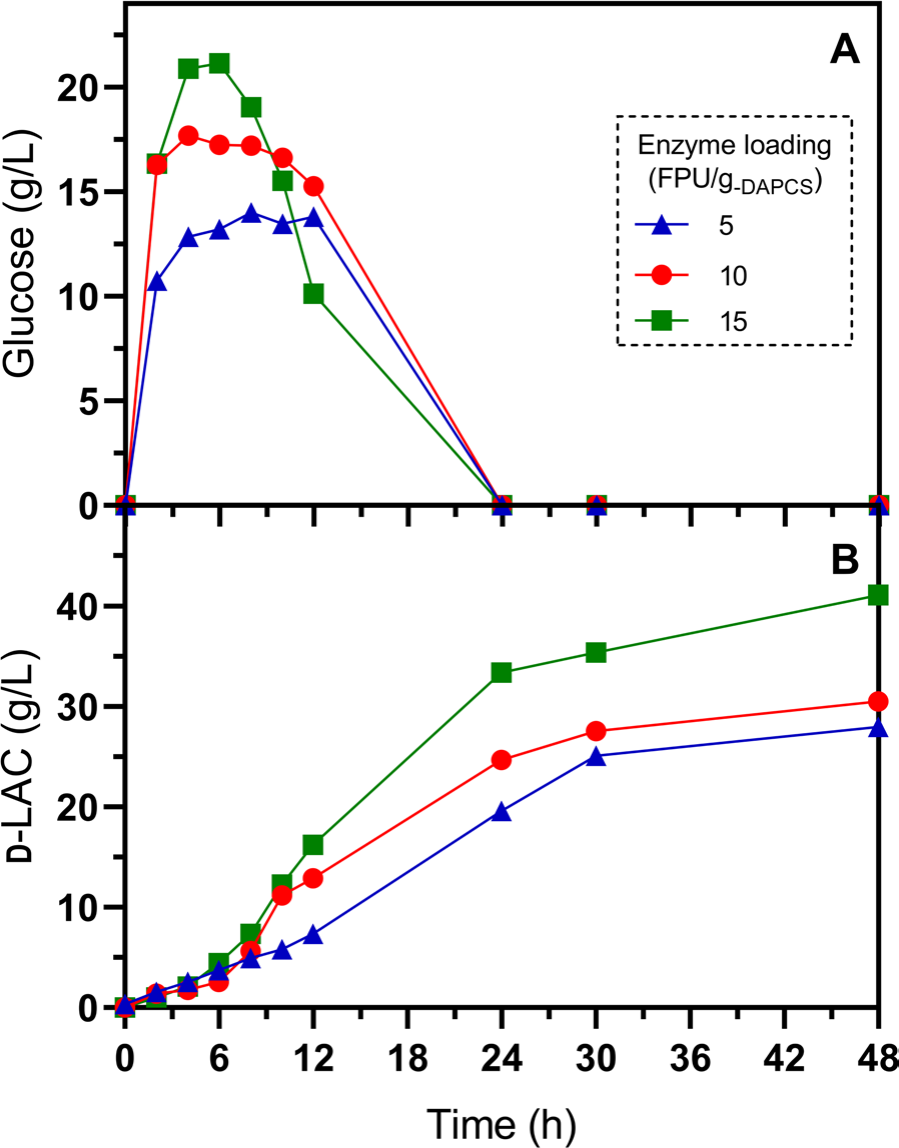
Simultaneous saccharification and fermentation of 10% (*w*/*w*) diluted acid-pretreated corn stover at different enzyme loadings, pH 6.3 and 47 °C. **A** Glucose release and consumption with an enzyme loading of 5, 10, and 15 FPU/g_-DAPCS_. **B** d-LAC production with an enzyme loading of 5, 10, and 15 FPU/g_-DAPCS_. Error bars in figures are shorter than the size of the symbol. These cultivations were performed in duplicate

After 24 h of SSF, the glucose concentration in the liquid phase approached zero, while d-LAC accumulated at concentrations of 44, 33, and 19 g/L under enzyme loadings of 15, 10, and 5 FPU/g_-PCS,_ respectively (Fig. 3). Subsequently, the rate of d-LAC accumulation decreased between 24 and 48 h, and at 48 h of SSF the d-LAC concentration was 60, 41, and 30 g/L, respectively. Accordingly, the rate of d-LAC production was 30% and 50% lower with 10 and 5 FPU/g_-DAPCS_ compared with 15 FPU/g_-DAPCS_ (Table 4). Therefore, we deduced that reducing enzyme loading had a detrimental effect on d-LAC production, indicating that under our experimental conditions, SSF of DAPCS did not allow enzyme quantity reduction. This trend was also observed even with SSF conducted at 10% solid loading (Fig. 5 and Table 4).

**Table 4:** Fermentation parameters of SSF of DAPCS biomass at 48 h (pH 6.3)

Enzyme activity (FPU/g_-DAPCS_)	Strain GT48 (47 °C)						Strain JU15 (45 °C)
DAPCS load (w/w)	5		10		15		15
Parameter	10%	15%	10%	15%	10%	15%	15%
Qp_48_ (g/L∙h)	0.58 (0.002)	0.61 ^****^ (0.001)	0.64 (0.002)	0.86 ^****^ (0.001)	0.86 (0.012)	1.23 ^****^ (0.002)	0.87 ^****^ (0.024)
Yp/s (g d-LAC/g glucan)	50.41% (0.139)	35.18% ^****^ (0.08)	55.03% (0.18)	49.38% ^****^ (0.04)	74.08% (1.035)	71.03%^**^(0.138)	49.96% ^****^ (1.383)
d-LAC_Total_ (g/L)	27.98 (0.077)	29.29 ^****^ (0.063)	30.54 (0.099)	41.11 ^****^ (0.037)	41.12 (0.575)	59.13 ^****^ (0.115)	41.6 ^****^ (1.151)

The volumetric productivity (Qp) is reported at 48 h. The global yield of d-LAC on substrate (Yp/s) was calculated in relation to the percentage of glucans present in the solid loading. The d-LAC titer corresponds to the final time of fermentation in AM1 minimal medium supplemented with 1 g/L tryptone and 0.5 g/L yeast extract. Values in parenthesis indicate standard deviation from duplicates. Asterisks represent significant statistical differences from a Student’s *t*-test performed between a) each DAPCS load at the same enzyme activity and b) Strain JU15 versus GT48 at the same DAPCS load and enzyme activity. ANOVA analysis between the same DAPCS load at different enzyme activity is statistically significant in all parameters (^****^)

In previous works, hemicellulose hydrolysates of sugar cane bagasse and corn stover containing glucose, xylose, and arabinose were fermented to d-LAC at 37 °C with the strain JU15 [27]. This process yielded 48 g/L and 58 g/L of d-LAC after 48 h with conversion yields of 1.3 g_d-LA_/g_sugars_ and 1.11 g_d-LA_/g_sugars_, and volumetric rates of 0.51 and 1.21 g/L/h, respectively. Additionally, JU15 was also used at 37 °C to ferment avocado seed hydrolysates containing glucose (40 g/L) and xylose (2 g/L) [29], resulting in the accumulation of 37 g/L of d-LAC at a rate of 1.6 g/L/h and a conversion yield of 94%. In another work, food waste hydrolysates containing glucose, fructose, and arabinose were also converted to d-LAC using the JU15 strain achieving a final titer of 22.66 g/L, a volumetric productivity of 0.73 g/L/h, and a conversion yield, from released reducing sugars of 99% [28]. Together, these results are similar to those obtained with strain GT48 for d-LAC production but under actual SSF of pretreated lignocellulosic biomass at 47 °C. Therefore, GT48 kept comparable metabolic capabilities to its progenitor JU15 despite its evolution at 48 °C.

**Fig. 6:**
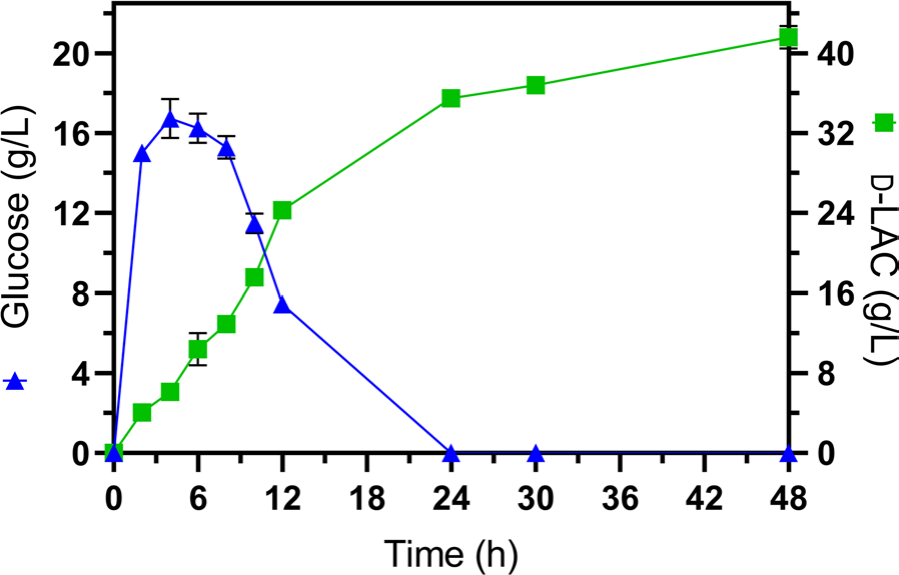
Simultaneous saccharification and fermentation of 15% (*w*/*w*) diluted acid-pretreated corn stover by parental *E. coli* strain JU15 at 15 FPU/g-_DAPCS_, pH 6.3 and 45 °C. Glucose release and consumption and d-LAC production. Error bars in the figures represent the standard deviation from duplicates

The comparison between SSF performed at 47 °C with 15% (*w*/*w*) solids loading using GT48 and SSF with JU15 at 45 °C and pH 6.3 reveal intriguing insights (Fig. 6 and Table 4). Initially, after 6 h, glucose and d-LAC reached concentrations of 17 g/L and 11 g/L, respectively. This translates to a 60% reduction in glucose and a sixfold increase in d-LAC than in SSF with GT48 at 47 °C (Fig. 3). In this sense, during the initial stage of cultivation, a low glucose concentration favors a higher glucose-to-LA conversion rate. These findings suggest that at 45 °C, metabolic activation for d-LAC fermentation in JU15 was favored over GT48 at 47 °C, while cellulase activity underwent a significant decline. Consequently, there was no discernible lag phase for d-LAC synthesis at 45 °C. However, after 24 h of SSF, d-LAC accumulation with JU15 at 45 °C was 22% lower than with GT48 at 47 °C (35 vs 45 g/L) (Figs. 3 and 6). Furthermore, after 48 h, d-LAC accumulation was 30% lower at 45 °C using JU15 compared to GT48 at 47 °C (42 vs 60 g/L). Notably, SSF with JU15 at 45 °C and 15 FPU/g_-DAPCS_ yielded results quite akin to experiments with GT48 at 47 °C but with 10 FPU/g_-DAPCS_. This highlights that performing SSF with JU15 at 45 °C and 15 FPU/g_-DAPCS_ adversely impacted enzymatic activity. Therefore, our findings lend support to the potential use of the thermo-adapted strain GT48 in SSF at temperatures close to the optimum for cellulases to diminish the reduction of enzymatic activity caused by decreased temperature necessary to conciliate with microbial growth requirements.

## Conclusions

A population of *E. coli* JU15 strain underwent successful adaptation to 48 °C in glucose-mineral media without aeration, pH 7, and a low supplementation of protein hydrolysates. Although GT48 could not sustain stable growth at 48 °C, it effectively accumulated d-LAC at 47 °C. During SSF at 15% (w/w) solids loading, 15 FPU/g-_DAPCS_, pH 6.3, and 47 °C, the strain GT48 achieved d-LAC concentrations comparable to those observed in various separated saccharification and fermentation processes reported previously. Therefore, the d-LAC production from pretreated corn stover biomass was established in SSF by a thermally evolved *E. coli* strain GT48.

## Data Availability

No datasets were generated or analysed during the current study.

## Abbreviations

ALE: Adaptive laboratory evolution
d-LAC: D-Lactic acid
DAPCS: Diluted acid-pretreated corn stover
FPU/mL: Filter paper unit per milliliter
FPU/g_-DAPCS_: Filter paper unit per gram of diluted acid pretreatment corn stover
NGMAC: Non-growing but metabolically active cells
SSF: Simultaneous saccharification and fermentation
